# Supplementary material for: Empirical methods for controlling false positives and estimating confidence in ChIP-Seq peaks
Source: BMC Bioinformatics. 2008 Dec 5;9:523. doi: 10.1186/1471-2105-9-523 (PMC2628906; doi:10.1186/1471-2105-9-523)
Supplement: Additional File 1 — A variety of html documents from the USeq web site detailing the available applications, their best usage, output file type descriptions, command line menus, etc. [file 1471-2105-9-523-S1.zip › USeqUserGuides/JavaDoc.zip!/JavaDoc/trans/main/package-tree.html]

trans.main Class Hierarchy


|  |  |  |
| --- | --- | --- |
| **Overview** \| **Package** \| Class \| Use \| **Tree** \| **Deprecated** \| **Index** \| **Help** | |  |
| **PREV**   **NEXT** | **FRAMES**    **NO FRAMES**     **All Classes** |  |


---


## Hierarchy For Package trans.main

**Package Hierarchies:**: All Packages

---

## Class Hierarchy

- java.lang.Object
  - trans.main.**BindingPeak** (implements java.io.Serializable)- trans.main.**BindingRegionGraphPrinter**- trans.main.**ChromSet**- trans.main.**ExportIntervalData**- trans.main.**FDRVsQValues**- trans.main.**FDRWindowConverter**- trans.main.**FindSubBindingRegions**- trans.main.**IntensityPrinter**- trans.main.**Interval** (implements java.lang.Comparable<T>, java.io.Serializable)- trans.main.**IntervalComparator** (implements java.util.Comparator<T>)- trans.main.**IntervalFilter**- trans.main.**IntervalGFFPrinter**- trans.main.**IntervalGraphPrinter**- trans.main.**IntervalMaker**- trans.main.**IntervalReportPrinter**- trans.main.**LoadChipSetIntervalOligoInfo**- trans.main.**LoadIntervalOligoInfo**- trans.main.**MakeAllWindows**- trans.main.**MergeWindowArrays**- trans.main.**MultiSGRPrinter**- trans.main.**MultiWindowIntervalMaker**- trans.main.**Oligo** (implements java.io.Serializable)- trans.main.**OligoIntensityPrinter**- trans.main.**OverlapCounter**- trans.main.**PeakPicker**- trans.main.**PosRegion** (implements java.lang.Comparable<T>)- trans.main.**RandomizeLabels**- trans.main.**ReplaceWindowScores**- trans.main.**SamChromosomeData**- trans.main.**ScanChip**- trans.main.**ScanChromosomes**- trans.main.**ScanChromosomesCNV**- trans.main.**ScanGenes**- trans.main.**ScoredGene** (implements java.lang.Comparable<T>)- trans.main.**ScoreIntervals**- trans.main.**SetNumberIntervalMaker**- trans.main.**SplitWindowArraysByChromosome**- trans.main.**SubWindow** (implements java.io.Serializable)- trans.main.**T2**- trans.main.**T2Parameter**- trans.main.**TestScanChip**- trans.main.**WilcoxonRankSumTest**- trans.main.**WilcoxonSample** (implements java.lang.Comparable<T>)- trans.main.**WilcoxonSamplePair** (implements java.lang.Comparable<T>)- trans.main.**WilcoxonSignedRankTest**- trans.main.**Window** (implements java.lang.Comparable<T>, java.io.Serializable)- trans.main.**WindowBlockMaker**- trans.main.**WindowBlockMakerTwoColor**- trans.main.**WindowComparator** (implements java.util.Comparator<T>)

---


|  |  |  |
| --- | --- | --- |
| **Overview** \| **Package** \| Class \| Use \| **Tree** \| **Deprecated** \| **Index** \| **Help** | |  |
| **PREV**   **NEXT** | **FRAMES**    **NO FRAMES**     **All Classes** |  |


---
